# A Five-Genes-Based Prognostic Signature for Cervical Cancer Overall Survival Prediction

**DOI:** 10.1155/2020/8347639

**Published:** 2020-03-25

**Authors:** Menghuang Zhao, Wenbin Huang, Shuangwei Zou, Qi Shen, Xueqiong Zhu

**Affiliations:** Department of Obstetrics and Gynecology, The Second Affiliated Hospital of Wenzhou Medical University, Wenzhou 325027, China

## Abstract

*Aims*. This study is aimed at identifying a prognostic signature for cervical cancer. *Main Methods*. The gene expression data and clinical information of cervical cancer and normal cervical tissues were acquired from The Cancer Genome Atlas and from three datasets of the Gene Expression Omnibus database. DESeq2 and Limma were employed to screen differentially expressed genes (DEGs). The overlapping DEGs among all datasets were considered the final DEGs. Then, the functional enrichment analysis was performed. Moreover, the Cox proportional hazards regression was performed to establish a prognostic signature of the DEGs. The Kaplan-Meier analysis was applied to test the model. Relationships between gene expression and clinicopathological parameters in cervical cancer, including age, HPV status, histology, stage, and lymph node metastasis, were analysed by the chi-square test. The somatic mutations of these prognostic genes were assessed through cBioPortal. The robustness of the model was verified in another two independent validation cohorts. *Key Findings*. In total, 169 overlapping upregulated genes and 29 overlapping downregulated genes were identified in cervical cancer compared with normal cervical tissues. Functional enrichment analysis indicated that the DEGs were mainly enriched in DNA replication, the cell cycle, and the p53 signalling pathway. Finally, a 5-gene- (ITM2A, DSG2, SPP1, EFNA1, and MMP1) based prognostic signature was built. According to this model, each patient was given a prognostic-related risk value. The Kaplan-Meier analysis showed that a higher risk was related to worse overall survival in cervical cancer, with an area under the receiver operating characteristic curve of 0.811 for 15 years. The validity of this model in the prediction of cervical cancer outcome was verified in another two independent datasets. In addition, our study also found that the low expression of ITM2A was associated with cervical adenocarcinoma. Interestingly, DSG2 was associated with the HPV status of cervical cancer. *Significance*. Our study constructed a prognostic model in cervical cancer and discovered two novel genes, ITM2A and DSG2, associated with cervical carcinogenesis and survival.

## 1. Introduction

Cervical cancer ranks as the fourth most common cancer in women worldwide [1]. Even in developed countries such as the United States, there were approximately 13 170 newly diagnosed cases of cervical cancer and 4 250 deaths in 2019 [2]. Although some advances have been made in the screening technology for cervical cancer and human papillomavirus vaccine applications, the survival rate remains poor [3]. Another critical problem with cervical cancer is the heterogeneity in clinical outcomes among individuals with the same disease [4]. Consequently, there is a great need to establish a molecular tool to help predict patient outcomes and outline individualized treatment plans.

With the impressive progress of the next-generation sequencing technology, various genetic alterations have been revealed, including the high-frequency mutations of genes and dysregulated signalling pathways in cancer [5]. Recently, numerous studies have shown the clinical importance of the messenger RNA expression in numerous cancers, including cervical cancer [6]. Messenger RNAs play pivotal roles in *diverse* physiological and pathological processes, such as differentiation, cell proliferation, development, apoptosis, and stress responses [7].

Transcriptome sequencing technologies (RNA-Seq) and microarray data provide an ideal platform for cancer genetic studies. The Gene Expression Omnibus (GEO) and The Cancer Genome Atlas (TCGA) repositories offer abundant cervical cancer sample resources, which may be helpful to explore reliable biomarkers. In the current study, gene expression in cervical cancer was profiled to identify critical genes predictive of patient outcomes. Through the differentially expressed gene (DEG) analysis and the Cox proportional hazards model, a signature based on the expression of several key genes was finally constructed as a prognostic signature for cervical cancer. This prognostic model could be used as an effective tool to predict the prognosis of patients with cervical cancer. The findings can also aid in identifying new therapeutic targets for cervical cancer.

## 2. Materials and Methods

### 2.1. Data Source

A high-throughput sequencing dataset and the clinical information of cervical cancer patients were downloaded from the TCGA website for the screening of prognostic signatures. Three microarray datasets (GSE6791 [8], GSE7803 [9], and GSE9750 [10]) were obtained from the GEO database. In total, 423 samples, including 378 cervical cancer samples and 45 normal cervical tissue samples, were collected in the present study.

### 2.2. Identification of DEGs

Raw count data were extracted from TCGA. The DESeq2 package [11] for R was used to normalize and identify DEGs. DEGs in cervical cancer samples compared with adjacent normal cervical tissues were obtained with the threshold of adjusted *P* value < 0.05 and absolute log2 − based fold change > 1. The Limma package [12] for R was used for the microarray data to identify DEGs. The thresholds of absolute log2 − based fold change > 1 and *false* *discovery* *rate* (FDR) < 0.05 were employed to screen out DEGs. The overlapping DEGs among all datasets were considered the final DEGs.

### 2.3. Functional Enrichment

The Gene Ontology (GO) resource provides scientific knowledge about the functions of genes from many different organisms [13, 14]. The GO enrichment analysis was performed by employing the online software Database for Annotation, Visualization, and Integration Discovery (DAVID, version 6.8) [15]. GO terms were composed of three categories, molecular function (MF), cellular component (CC), and biological process (BP). Pathway enrichment was analysed by The Kyoto Encyclopedia of Genes and Genomes (KEGG) pathway database [16], which consists of a collection of biochemical pathway diagrams of reaction networks and molecular interactions. *P* < 0.05 was considered a significant difference.

### 2.4. Prognostic Signature Construction and Evaluation

Expression profiles with fragments per kilobases per million values were downloaded from TCGA, and the log2 transformation was performed. The prognostic value of each DEG was analysed by the univariate Cox proportional hazards regression analysis using the survival package [17] for R. DEGs that were significantly related to overall survival (*P* < 0.05) were retained for further analysis. Next, the multivariable Cox proportional hazards stepwise regression with backward selection in SPSS (version 18.0) was used to establish a prognostic model. A prognostic signature was built based on the linear weighted combination of the gene expression values. The weight of each gene was the regression coefficient (*β*) identified from the multivariate Cox proportional hazards regression analysis. The prognostic risk score for predicting overall survival (OS) was as follows: Risk score = *Σβ*i × expGenei. According to the median risk score, patients were divided into low- and high-risk groups. Then, the prognostic signature model was examined by the Kaplan-Meier analysis. To assess the predictive accuracy of the prognostic signature for cervical cancer outcome, a receiver operating characteristic (ROC) curve was plotted by the survivalROC package for R.

### 2.5. Analysis of Clinicopathological Parameters in Cervical Cancer

The expression levels of the prognostic genes and clinicopathological parameters of cervical cancer patients, including age, HPV status, histology, stage, and lymph node metastasis, were downloaded from TCGA. Only samples with relevant clinical information were included. The patients were classified into low and high expression groups according to the median gene expression value. The relationship between gene expression and clinicopathological parameters in cervical cancer was analysed by the chi-square test. *P* < 0.05 was considered to indicate a statistically significant difference.

### 2.6. Prognostic Gene Mutations

The somatic mutations of the prognostic genes in cervical cancer patients, including missense mutations, truncation mutations, fusions, amplifications, and deep deletions, were assessed through cBioPortal [18].

### 2.7. Validation of the Robustness of the Prognostic Signature

The robustness of the prognostic signature for predicting survival in cervical cancer patients was evaluated in the validation cohorts GSE52903 and GSE39001 [19, 20]. The differential expression levels of the corresponding genes were compared by Student's *t*-test. The Kaplan-Meier analysis was applied to confirm the prognostic signature of the prediction model.

### 2.8. Prognostic Values of Clinical Parameters and Risk Score in Cervical Cancer

Furthermore, patients with all clinical information, including age, HPV status, histology, stage, and lymph node metastasis, were collected from TCGA. Then the univariate Cox proportional hazards regression and multivariable Cox proportional hazards regression were used to analyse the prognostic values of clinical parameters and risk score in cervical cancer. *P* < 0.05 was considered to indicate a statistically significant difference.

## 3. Results

### 3.1. Identification of Differentially Expressed Genes between Cervical Cancer and Normal Cervical Tissue

**Figure 1:**
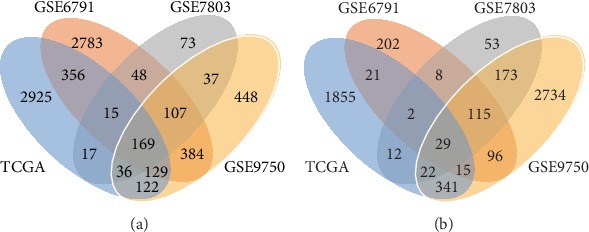
Venn diagrams showing the overlapping DEGs in the four datasets. (a) Upregulated DEGs. (b) Downregulated DEGs.

In the TCGA dataset, compared with normal cervical samples, 3 769 genes were identified to be significantly upregulated, while 2 297 genes were discovered to be significantly downregulated in cervical cancer. For the GSE6791 dataset, the differential expression analysis between cervical cancer and normal cervical samples identified 3 991 upregulated DEGs and 488 downregulated DEGs. For the GSE7803 and GSE9750 datasets, 502 upregulated and 414 downregulated DEGs and 1 432 upregulated and 3 525 downregulated DEGs were found in cervical cancer, respectively. Altogether, compared with the normal cervical samples, 169 upregulated DEGs and 29 downregulated DEGs in cervical cancer overlapped among the three microarray datasets and the TCGA dataset (Figure 1).

### 3.2. Functional Enrichment in Cervical Cancer

**Figure 2:**
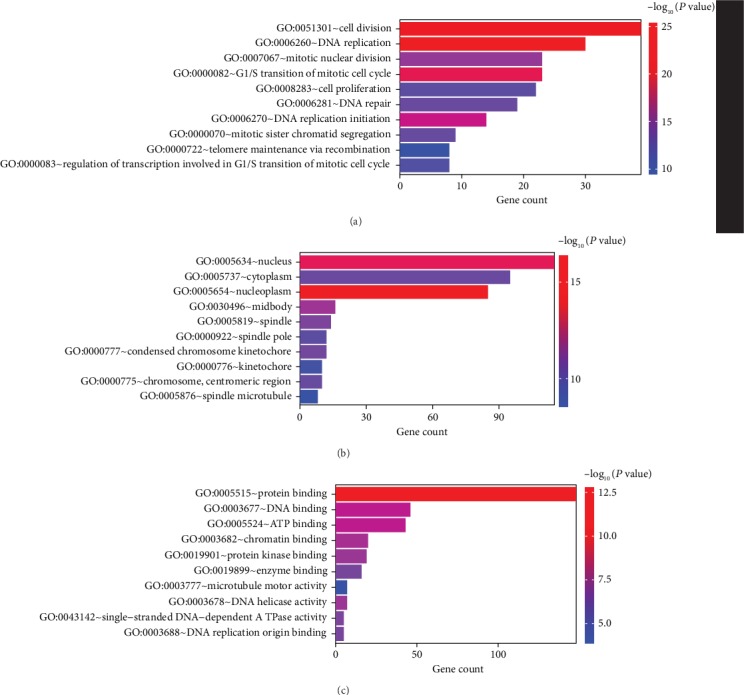
The results of the GO enrichment analysis of DEGs. (a) Biological processes. (b) Cellular components. (c) Molecular functions.

GO enrichment showed that the DEGs were primarily related to biological process functions, which included DNA replication (*P* = 2.23 × 10^−27^), cell division (*P* = 1.12 × 10^−26^), and G1/S transition of the mitotic cell cycle (*P* = 2.05 × 10^−22^). The GO results of the cellular compartment category revealed that the DEGs were mainly enriched in the nucleoplasm (*P* = 1.04 × 10^−20^), nucleus (*P* = 6.42 × 10^−17^), and midbody (*P* = 8.89 × 10^−12^). In terms of molecular functions, the DEGs were involved in protein binding (*P* = 8.21 × 10^−14^), ATP binding (*P* = 1.54 × 10^−8^), and DNA binding (*P* = 1.57 × 10^−8^). The top 10 GO terms are shown in Figure 2.

**Figure 3:**
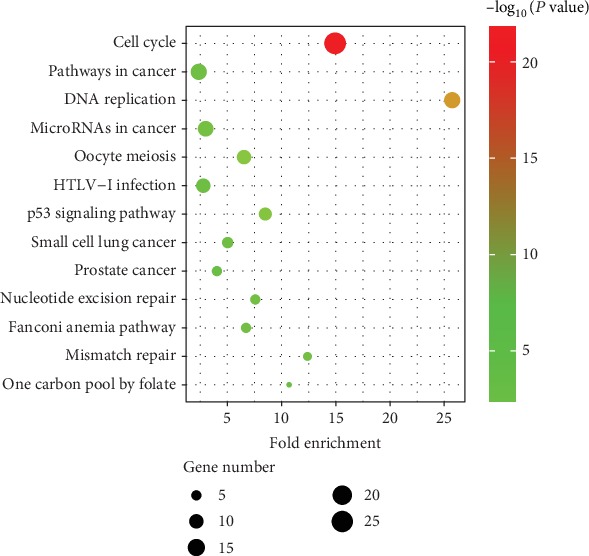
The results of the KEGG enrichment analysis of DEGs.

The KEGG pathway enrichment showed that the DEGs were significantly enriched in the DNA replication, cell cycle, and p53 signalling pathways (Figure 3).

### 3.3. Prognostic Signature Construction and Evaluation

The DEGs were further analysed to identify survival-related genes of cervical cancer through the univariate Cox proportional hazards analysis. The analysis identified 8 upregulated DEGs that were significantly related to poor overall survival in the TCGA cohort and 3 downregulated DEGs that were related to good overall survival (*P* < 0.05). In total, 11 survival-related DEGs were found.

**Figure 4:**
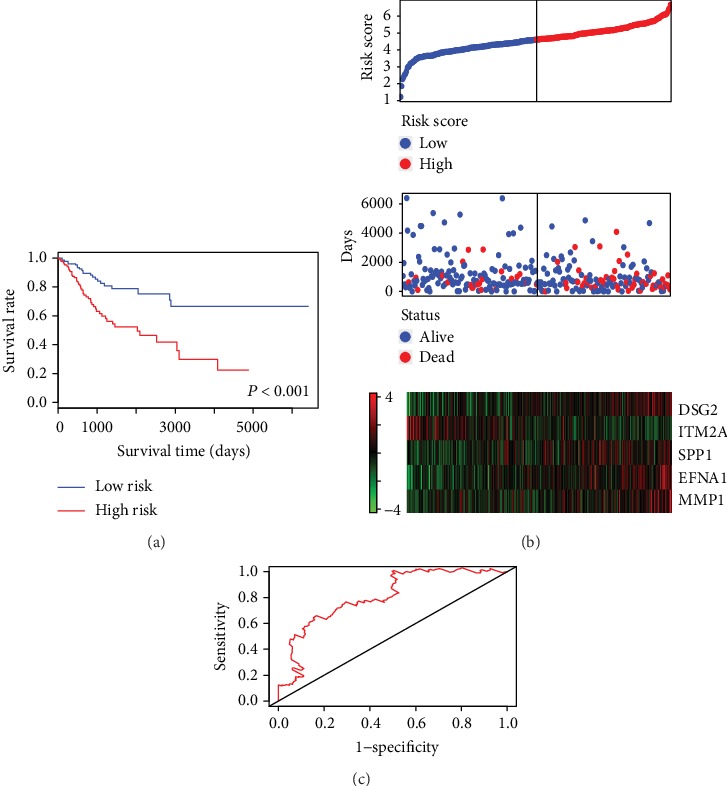
Prognostic evaluation of the 5-gene signature in cervical cancer patients. (a) The Kaplan-Meier risk survival curve analysis of overall survival in cervical cancer patients. (b) The distributions of the risk score, survival status, and gene expression levels of the patients. (c) ROC curve analysis of the 5-gene signature.

Then, the multivariable Cox proportional hazards analysis was performed to build a prognostic model. Five genes, ITM2A, DSG2, SPP1, EFNA1, and MMP1, which showed a significant *P* value, were selected (Table 1). A linear model was calculated with the risk score = (–0.308 × ITM2A) + (0.341 × DSG2) + (0.172 × SPP1) + (0.392 × EFNA1) + (0.116 × MMP1). The risk score was calculated for each patient in the TCGA dataset. According to the median risk score, the patients were divided into high- and low-risk groups. The Kaplan-Meier analysis was conducted and showed that the high-risk patient group had a markedly shorter overall survival than the low-risk patient group (Figure 4(a)). The distributions of the risk score, survival status, and gene expression levels of the individual patients are shown in Figure 4(b). Furthermore, the ROC analysis was performed to evaluate the predictive accuracy of the prognostic model. The area under the curve (AUC) for the 10-year prediction was 0.762. The AUC for the 15-year prediction was up to 0.811 (Figure 4(c)).

### 3.4. Analysis of Clinicopathological Parameters in Cervical Cancer

The associations between the expression of the 5 prognostic genes and the clinicopathological parameters of patients with cervical cancer were determined. As presented in Table 2, ITM2A expression was associated with the histology of cervical cancer. Cervical cancer patients with a low expression of ITM2A were more likely to have adenocarcinoma or adenosquamous carcinoma, while cervical cancer patients with a high expression of ITM2A tended to have squamous carcinoma. However, ITM2A exhibited no association with age, HPV status, stage, or lymph node metastasis in cervical cancer.

The relationships between DSG2 expression and different clinicopathological parameters in cervical cancer are demonstrated in Table 3. Interestingly, DSG2 expression was related to HPV status. All cervical cancer patients with a high expression of DSG2 were HPV positive. However, approximately 9.1% of patients with a low expression of DSG2 were HPV negative. DSG2 expression was not correlated with other clinicopathological parameters in cervical cancer patients.

Tables 4 and 5 show that EFNA1 and MMP1 exhibited no association with any clinicopathological parameters in cervical cancer patients.

Table 6 shows the results of SPP1 in cervical cancer. SPP1 was found to be related to the age and histology of cervical cancer patients. Patients with high SPP1 expression were older than those with low SPP1 expression. The low expression group tended to have adenocarcinoma-type cervical cancer.

### 3.5. Prognostic Gene Mutations

**Figure 5:**
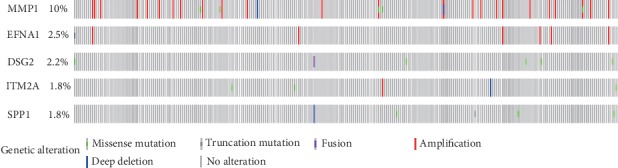
Somatic mutations of the five genes contained in the prognostic signature.

The somatic mutations of the five prognostic genes were further explored in this study (Figure 5). In cervical cancer, 10% of patients showed genetic alterations in MMP1. EFNA1 mutation occurred in 2.5% of cervical cancer patients. Amplification was the most common mutation in these two genes. The mutation rates of DSG2, SPP1, and ITM2A were 2.2%, 1.8%, and 1.8%, respectively. Deletion could be seen in ITM2A. These mutations might contribute to the aberrant expression of the corresponding genes.

### 3.6. Validation of the Robustness of the Prognostic Signature

**Figure 6:**
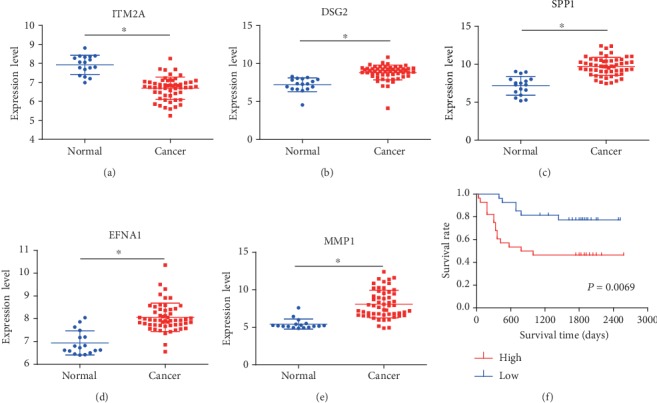
The expression level and prognostic value of the five genes in the GSE52903 validation dataset. Differential expression levels of the five genes between cervical cancer and normal cervical tissues: (a) ITM2A, (b) DSG2, (c) SPP1, (d) EFNA1, and (e) MMP1. (f) The Kaplan-Meier risk survival curve analysis of overall survival in cervical cancer patients.

The robustness of the prognostic signature for predicting survival in cervical cancer patients was evaluated in the validation cohorts GSE52903 and GSE39001. The GSE52903 dataset was composed of 17 normal cervical tissues and 55 cervical cancer samples. First, the differential expression level of the five prognostic biomarkers between normal cervical tissue and cervical cancer samples was tested. ITM2A was downregulated in cervical cancer compared with normal cervical samples, and the four other genes (DSG2, SPP1, EFNA1, and MMP1) were markedly upregulated in cervical cancer, which was consistent with the results above (Figures 6(a)–6(e)). Then, the risk scores of individual cervical cancer patients were calculated according to the prognostic linear model shown above. The patients were divided into high- and low-risk groups. The Kaplan-Meier results demonstrated that a high-risk score was associated with shorter overall survival, which confirmed the results discovered above (Figure 6(f)).

**Figure 7:**
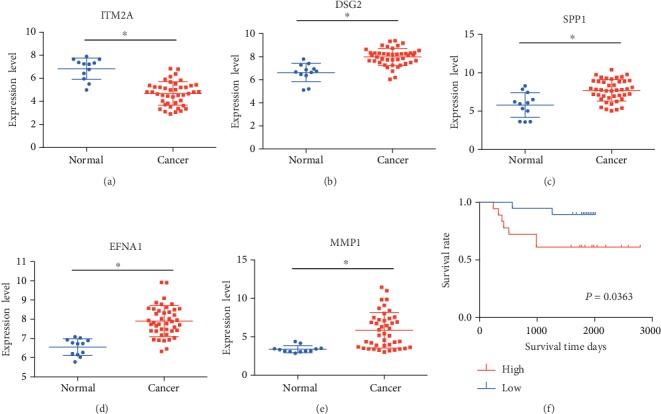
The expression level and prognostic value of the five genes in the GSE39001 validation dataset. Differential expression levels of the five genes between cervical cancer and normal cervical tissues: (a) ITM2A, (b) DSG2, (c) SPP1, (d) EFNA1, and (e) MMP1. (f) The Kaplan-Meier risk survival curve analysis of the overall survival in cervical cancer patients.

The GSE39001 dataset consisted of 12 normal cervical tissues and 43 cervical cancer samples. The above results were further verified in the GSE39001 dataset (Figure 7).

### 3.7. Prognostic Values of Clinical Parameters and Risk Score in Cervical Cancer

Furthermore, the prognostic values of clinical parameters of patients with cervical cancer were also analysed (Table 7). The univariate Cox proportional hazards regression analysis showed that lymph node metastasis and risk score were associated with overall survival. However, the multivariable Cox proportional hazards regression revealed that only the risk score was an independent prognostic indicator of cervical cancer survival.

## 4. Discussion

Cervical cancer is one of most lethal malignancies in women, causing a heavy burden to families and the world [21]. The accurate prediction of cervical cancer outcomes is of great importance for therapy method selection and prognosis improvement. In the present study, four independent datasets were used to identify DEGs between cervical cancer and normal cervical tissue. In total, 198 DEGs, including 29 downregulated and 169 upregulated genes, were identified. To better understand the mechanism of cervical tumourigenesis, enrichment analysis of the DEGs was performed. In terms of GO enrichment, the DEGs were mainly enriched in DNA replication, cell division, and G1/S transition of the mitotic cell cycle. The KEGG pathway results showed that the DEGs were mainly involved in DNA replication and the cell cycle. These findings were consistent with the fact that DNA replication and cell cycle regulation play important roles in the development of cancer [22].

To identify survival-associated DEGs, univariate Cox proportional hazards regression was performed. In total, 11 survival-related DEGs were found. Then, by stepwise selection, 5 independent prognostic mRNAs (SPP1, EFNA1, MMP1, ITM2A, and DSG2) were selected and combined to construct a prognostic signature. The prognostic model based on these five genes showed high efficiency in distinguishing good versus poor outcomes for cervical cancer patients. According to the Hosmer-Lemeshow classification method, areas under the ROC curve of 0.80 to 0.90 are considered to be excellent [23]. The areas under the ROC curve of this model reached 0.811 for the 15-year prediction. The robustness of the prognostic signature was confirmed in two independent cohorts. In the clinic, this prognostic signature might serve as a predictive indicator of cervical cancer survival.

SPP1 is a secreted glycophosphoprotein of the SIBLING family that plays a critical role in physiological and pathophysiological processes [24]. Elevated expression of SPP1 has been observed in multiple cancers, including colon cancer, lung cancer, prostate cancer, breast cancer, ovarian cancer, multiple myeloma, acute myeloid leukaemia, and chronic myeloid leukaemia [25–27]. SPP1 expression was also higher in cervical cancer [28]. In the study by Cho, SPP1 had a 50.6% sensitivity and 95.0% specificity as a diagnostic biomarker for cervical cancer [29]. Higher expression of SPP1 was strongly related to worse disease-free survival and overall survival in patients with cervical cancer [29]. The present findings were consistent with those of previous studies.

EFNA1, a glycosyl-phosphatidylinositol-anchored ligand, binds to its receptor EPHA2 to promote autophosphorylation [30, 31]. Autophosphorylation then triggers downstream signalling to regulate cell migration and growth [30]. EFNA1, a tumour-related gene, was significantly upregulated in numerous tumours, including cervical cancer [32–36]. EFNA1 expression was reported to be related to deep invasion, parametrial invasion, tumour size, and outcome in cervical carcinoma [36, 37]. Our study also observed the high expression of EFNA1 in cervical cancer, and its expression was related to poor overall survival.

Matrix metalloproteinase-1 (MMP1) functions as an enzyme in the degradation of the extracellular matrix in both normal physiological processes and disease processes [38]. Growing evidence has shown that MMP1 plays critical roles in tumourigenesis and cancer metastasis [39]. Some studies have observed the upregulation of MMP1 in cervical cancer [40]. Knockdown of MMP1 could inhibit cervical cancer cell invasion, migration, and proliferation via epithelial-mesenchymal transition [41]. MMP1 was correlated with lymph node metastasis and indicated unfavourable survival in cervical cancer [41]. The present study supported previous reports by showing that the high expression of MMP1 was significantly related to poor overall survival in cervical cancer.

ITM2A is a type II integral membrane protein containing a BRICHOS domain [42, 43]. Some studies supported that ITM2A was associated with cell differentiation, including chondrogenesis, odontogenesis. and myogenesis stages [44–46]. A previous study found that ITM2A was markedly downregulated in invasive ovarian carcinomas compared with normal ovarian tissues [47]. In addition, the loss of ITM2A was significantly correlated with higher FIGO stage, recurrence, chemoresistance, and poorer prognosis in ovarian cancer [47]. The expression of ITM2A induced G2/M cell cycle arrest and inhibited ovarian cancer cell growth by decreasing the expression of cyclin B1, p-CDC2, CDC2, and CDC25C [47]. All of the above results indicated that ITM2A might serve as a tumour suppressor in ovarian cancer. However, there has been no paper about the expression and prognostic value of ITM2A in cervical cancer. In this study, ITM2A was shown to be downregulated and related to good outcomes in cervical cancer. Different expression levels of ITM2A were also associated with the specific histological phenotype of cervical cancer. Cervical cancer patients with a low expression of ITM2A were more likely have adenocarcinoma or adenosquamous carcinoma, while cervical cancer patients with a high expression of ITM2A tended have squamous carcinoma. Compared with squamous cell carcinoma, cervical adenocarcinoma has a worse outcome [48, 49]. Our study found that the low expression of ITM2A was associated with poor prognosis and cervical adenocarcinoma. In clinical practice, ITM2A might be used as an indicator for the classification of cervical cancer, and cervical cancer patients with low ITM2A expression could be treated more aggressively. Of course, this finding still needs further investigation.

Intercellular adhesion plays an important role in the maintenance of multicellular structures and cell-cell signal transmission [50]. Desmosomes are intercellular connections that provide strong adhesion strength, and the dysregulation of desmosome components may lead to cancer progression by altering cellular signalling pathways [51]. DSG2 is a transmembrane desmosomal cadherin [52]. DSG2 has different functions in various cancers and can act as either an oncogene or a tumour suppressor. Several investigators reported high DSG2 expression in melanomas, human epithelial squamous cell cancer, basal cell cancer, non-small cell lung cancer, and colonic adenocarcinoma [52–55]. However, DSG2 was downregulated in prostate carcinoma, pancreatic tumours, and diffuse-type gastric cancer [56–58]. DSG2 was reported to be involved in regulating cell proliferation and tumourigenesis in cancers. Cai et al. [54] revealed that the knockdown of DSG2 expression led to the growth inhibition of non-small cell lung cancer cells and G1 phase arrest. Kamekura et al. [55] found that the loss of DSG2 inhibited cell proliferation through EGFR signalling in colon cancer. Vessel formation was attenuated in the DSG2 loss strain of mice [59]. Tan et al. [53] found that DSG2 might play crucial roles in regulating vasculogenic mimicry activity in human melanoma, which is another important method of blood supply in tumours. DSG2 also served as a poor prognostic indicator in melanoma [53]. There have been no reports about DSG2 in cervical cancer. In this study, the upregulation of DSG2 was observed in cervical cancer and indicated a poor outcome. Notably, DSG2 was associated with the HPV status of cervical cancer. These results suggested that DSG2 may be involved in HPV-induced cervical cancer. However, the involvement of the synergistic effects of DSG2 and HPV on cervical carcinogenesis needs further study.

## 5. Conclusion

In conclusion, the present study combined 5 microarray datasets with an RNA-Seq dataset and constructed and validated a 5-gene mRNA expression-based signature, which may serve as an independent indicator of cervical cancer survival. The prognostic values of SPP1, EFNA1, and MMP-1 in cervical cancer have been investigated in previous studies. Similar results were also found in our study. It is worth noting that ITM2A and DSG2 are two novel genes that have never been studied in cervical cancer. Furthermore, ITM2A was related to different histological phenotypes of cervical cancer. DSG2 was associated with the HPV status of cervical cancer. These findings lead to a better understanding of the underlying mechanisms of cervical cancer and help lay a foundation for making precise individual clinical treatment decisions. However, the present findings need further verification in future studies. Knockdown and overexpression of these five genes in cell and animal models could be performed to verify the carcinogenic functions of these genes in cervical cancer.

## Figures and Tables

**Table 1:** The multivariate Cox regression analysis of overall survival.

Gene symbol	Coefficient	Hazard ratio (HR)	*P* value
ITM2A	–0.308	0.735	0.042
DSG2	0.341	1.407	0.018
SPP1	0.172	1.187	0.011
EFNA1	0.392	1.479	0.012
MMP1	0.116	1.123	0.015

**Table 2:** Association between ITM2A expression and the clinicopathological parameters of cervical cancer patients.

Clinicopathological parameters		Low expression	High expression	*P* value
Age	<50 years	91	89	0.708
	>50 years	59	63	

HPV status	Negative	6	3	0.430
	Positive	80	89	

Histology	Adenocarcinoma	20	11	0.016^∗^
	Adenosquamous	3	0	
	Squamous	63	81	

Stage	I-II	63	78	0.138
	III-IV	20	14	

Lymph node metastasis	Positive	15	18	0.572
	Negative	35	53	

^∗^ *P* < 0.05.

**Table 3:** Association between DSG2 expression and the clinicopathological parameters of cervical cancer patients.

Clinicopathological parameters		Low expression	High expression	*P* value
Age	<50 years	82	98	0.061
	>50 years	69	53	

HPV status	Negative	9	0	0.016^∗^
	Positive	90	79	

Histology	Adenocarcinoma	18	13	0.373
	Adenosquamous	3	0	
	Squamous	78	66	

Stage	I-II	78	63	0.803
	III-IV	18	16	

Lymph node metastasis	Positive	15	18	0.054
	Negative	57	31	

^∗^ *P* < 0.05.

**Table 4:** Association between EFNA1 expression and the clinicopathological parameters of cervical cancer patients.

Clinicopathological parameters		Low expression	High expression	*P* value
Age	<50 years	88	92	0.543
	>50 years	64	58	

HPV status	Negative	7	2	0.171
	Positive	82	87	

Histology	Adenocarcinoma	15	16	0.331
	Adenosquamous	3	0	
	Squamous	71	73	

Stage	I-II	70	71	0.730
	III-IV	18	16	

Lymph node metastasis	Positive	18	15	0.738
	Negative	45	43	

**Table 5:** Association between MMP1 expression and the clinicopathological parameters of cervical cancer patients.

Clinicopathological parameters		Low expression	High expression	*P* value
Age	<50 years	83	97	0.075
	>50 years	69	53	

HPV status	Negative	6	3	0.391
	Positive	78	91	

Histology	Adenocarcinoma	18	13	0.375
	Adenosquamous	2	1	
	Squamous	64	80	

Stage	I-II	68	73	0.460
	III-IV	14	20	

Lymph node metastasis	Positive	12	21	0.095
	Negative	47	41	

**Table 6:** Association between SPP1 expression and the clinicopathological parameters of cervical cancer patients.

Clinicopathological parameters		Low expression	High expression	*P* value
Age	<50 years	101	79	0.010^∗^
	>50 years	50	72	

HPV status	Negative	3	6	0.494
	Positive	86	83	

Histology	Adenocarcinoma	23	8	0.002^∗^
	Adenosquamous	0	3	
	Squamous	66	78	

Stage	I-II	74	67	0.237
	III-IV	14	20	

Lymph node metastasis	Positive	13	20	0.17
	Negative	47	41	

^∗^ *P* < 0.05.

**Table 7:** Prognostic values of clinical parameters and risk score in cervical cancer.

Variables		Cases	Univariate Cox		Multivariable Cox	
			HR	*P* value	HR	*P* value
Age	<50 years	72				
	>50 years	48	0.794	0.643		

HPV status	Positive	7				
	Negative	113	0.044	0.460		

Histology	Squamous	94				
	Adenocarcinoma	23	1.277	0.670		
	Adenosquamous	3	<0.001	0.985		

Stage	I-II	102				
	III-IV	18	0.442	0.429		

Lymph node metastasis	Positive	87				
	Negative	33	0.312	0.020^∗^	0.819	0.735

Risk score			4.138	<0.001^∗^	3.845	0.001^∗^

^∗^ *P* < 0.05, HR: hazards ratio.

## Data Availability

Expression data and clinical information of cervical cancer and normal cervical samples analyzed in this study may be acquired from the TCGA (http://cancergenome.nih.gov/) and GEO (http://www.ncbi.nlm.nih.gov/geo/).

## Supplementary Materials

Supplementary Materials Table SI: differentially expressed genes.
