# Supplementary material for: A Five-Genes-Based Prognostic Signature for Cervical Cancer Overall Survival Prediction
Source: Int J Genomics. 2020 Mar 25;2020:8347639. doi: 10.1155/2020/8347639 (PMC7136791; doi:10.1155/2020/8347639)
Supplement: Supplementary Materials — Table SI: differentially expressed genes. [file 8347639.f1.docx]

Table SI. Differentially expressed genes

| Differentially expressed genes | | | | |
| --- | --- | --- | --- | --- |
| DBF4 | ORC6 | NCF2 | NUSAP1 | AR |
| DCN | ESR1 | CDC20 | KIF23 | EFNA1 |
| BRCA1 | CDC45 | STMN1 | IFI44 | ALCAM |
| TACC3 | CDC7 | NEK2 | KIF11 | CDK1 |
| ATP2C1 | GADD45B | CENPF | CEP55 | ARL6IP1 |
| HMGB3 | MCM5 | KIF14 | DNA2 | PDGFD |
| DSG2 | POLE2 | SPP1 | FANCI | SHCBP1 |
| POLQ | CDKN3 | ACYP1 | ABCA8 | KRT19 |
| MPHOSPH9 | VRK1 | HELLS | PLK4 | RRM2 |
| LAMC2 | GINS1 | EGR1 | KIF2C | HSPA6 |
| PARP12 | POLA1 | TMPO | DTL | CKS1B |
| CDH3 | NUP93 | ZSCAN18 | SLIT2 | RSRC1 |
| MCM10 | OIP5 | TNFSF10 | PLA2G7 | CCNE2 |
| MTHFD2 | MCM4 | PLAU | CDKN2A | ZNF135 |
| ASPM | CCNE1 | ZWINT | MKI67 | TYMS |
| NAV3 | MET | CDKN2C | HSPB8 | RMI1 |
| MAOB | NUDT1 | HJURP | SPARCL1 | APOBEC3B |
| TGFBR3 | EZH2 | CKS2 | BUB1B | SHMT2 |
| HLTF | LHX2 | CSE1L | DONSON | NDN |
| TFRC | CXCL12 | IFI6 | RACGAP1 | KNTC1 |
| SMC1A | NCAPG | DLGAP5 | SNRNP25 | USP18 |
| HMMR | FOXM1 | SYNGR3 | PRKCI | KIF18B |
| MCM2 | RAD51AP1 | GAD1 | AIM2 | ISG15 |
| GSDMB | RFC5 | E2F8 | GMPS | FANCA |
| TIPIN | TIMELESS | DNMT1 | TOPBP1 | HIST1H1C |
| GTSE1 | FBXO5 | SLC6A8 | RFC4 | COL14A1 |
| MOCOS | E2F3 | GINS2 | RPL39L | PLSCR1 |
| MCM6 | GMNN | IDO1 | CDC25A | APOD |
| SPAG5 | TTK | PSMC3IP | HMGB2 | SYCP2 |
| LAMP3 | KIF20A | TOP2A | MAD2L1 | GREB1 |
| ITM2A | LMNB1 | NUP210 | CITED2 | MMP1 |
| RBL1 | SMC4 | PCNA | PTTG1 | RAD54B |
| NDC80 | BCHE | NASP | CENPN | PEG3 |
| RAD54L | ECT2 | RFC3 | MCM7 | WDHD1 |
| AURKA | C3orf52 | ATP13A3 | TUBA1A | ARC |
| TPX2 | STAT1 | CCNB1 | CHAF1A | PRC1 |
| RBM41 | IGFBP5 | IGF2BP3 | PHYHIP | MICB |
| BIRC5 | CACYBP | ACTL6A | FEN1 | FANCG |
| KIF4A | WRAP73 | SMC2 | SHOX2 | AQP1 |
| LYN | MMP12 | FOXD1 |  |  |
